# Isoalantolactone exhibits activity against *Echinococcus multilocularis* associated with mitochondrial perturbation and changes in the lesion microenvironment

**DOI:** 10.3389/fphar.2026.1949982

**Published:** 2026-10-07

**Authors:** Wei Ma, Chuanchuan Liu, Haining Fan

**Affiliations:** 1 Department of Cardiovascular Surgery, Qinghai University Affiliated Hospital (Clinical Medical College), Xining, Qinghai Province, China; 2 Central Laboratory, Qinghai University Affiliated Hospital (Clinical Medical College), Xining, Qinghai Province, China; 3 General Surgery, Qinghai University Affiliated Hospital (Clinical Medical College), Xining, Qinghai Province, China

**Keywords:** alveolar echinococcosis, antiparasitic therapy, Echinococcus multilocularis, host immune response, isoalantolactone, lesion microenvironment, mitochondrial perturbation, natural products

## Abstract

**Background:**

Alveolar echinococcosis (AE) is a severe parasitic disease caused by *Echinococcus multilocularis*. Current therapy relies mainly on benzimidazole drugs and is largely parasitostatic, highlighting the urgent need for new candidate compounds with improved efficacy. We hypothesized that isoalantolactone (ISO) exerts direct anti-*Echinococcus multilocularis* activity and evaluated whether ISO treatment was associated with changes in the lesion microenvironment and host immune responses during AE progression *in vivo*.

**Methods:**

*In vitro* experiments were conducted using isolated *Echinococcus multilocularis* protoscoleces, metacestode vesicles, and germinal layer cells, with assessments of protoscolex survival, phosphoglucose isomerase release, and germinal cell viability. Scanning and transmission electron microscopy, mitochondrial membrane potential detection, molecular docking, and protein expression analysis were also performed. *In vivo* efficacy was verified using a murine intraperitoneal infection model treated with ISO or albendazole, combined with evaluations of lesion burden, histopathology, safety indicators, immune cell subsets, and cytokine levels.

**Results:**

ISO promoted phosphoglucose isomerase release, with a half-maximal effective concentration of 16.36 ± 3.24 μM, and reduced germinal cell viability, with a half-maximal inhibitory concentration of 17.46 ± 2.25 μM. It exerted dose- and time-dependent parasiticidal effects, achieving complete protoscolex death within 1–5 days at concentrations of 40 μM and 10 μM, respectively. In infected mice, ISO reduced lesion burden and spleen index and was associated with parasite structural damage, mitochondrial abnormalities, and changes in fibrosis-, inflammation-, and immune-related readouts under the present experimental conditions, with no marked abnormalities in most of the evaluated short-term safety parameters.

**Conclusion:**

ISO shows anti-*Echinococcus multilocularis* activity *in vitro* and suppresses AE progression *in vivo*. Its activity was associated with parasite structural injury, mitochondrial membrane potential depolarization, and changes in the lesion microenvironment and host immune responses. Further preclinical evaluation of ISO as a candidate compound against AE is warranted.

## 1 Introduction

Echinococcosis is an important zoonotic disease caused by parasites of the genus *Echinococcus*. The main clinical types include *cystic echinococcosis* (CE) and *alveolar echinococcosis* (AE). The liver is the most commonly affected organ, although lesions can also involve the lungs, brain, and other organs (World Health Organization [WHO], 2021; McManus et al., 2003). Compared with CE, which often presents as cystic lesions with relatively slow progression and clearer boundaries, AE is more invasive and infiltrative. AE often shows a “tumor-like” growth pattern, with unclear boundaries, easy involvement of blood vessels and bile ducts, and potential for distant dissemination (WHO, 2021). AE often lacks specific manifestations at the early stage and is therefore commonly diagnosed at an advanced stage in clinical practice. As a result, curative resection is difficult. Long-term drug maintenance therapy is required, and the risks of recurrence and death are significantly increased (McManus et al., 2012; Wen et al., 2019). China remains one of the major endemic regions for AE, with transmission concentrated predominantly in western China, particularly on the Tibetan Plateau. A national population-based survey reported an overall AE prevalence of 0.09% in China, with the highest provincial prevalence observed in Qinghai (0.43%), followed by Sichuan (0.28%) and Tibet (0.19%); highly endemic areas were mainly clustered around the junction of Qinghai, Tibet, and Sichuan (Ma et al., 2023). More recent nationwide surveillance data further documented 11,465 reported AE cases in China between 2004 and 2022, with the Tibetan Plateau showing the highest average annual AE case rate (46.95 cases per million population) (Wang X et al., 2025). These findings highlight the continuing public health importance of AE in western China and the need for effective therapeutic alternatives. Effective drug intervention is therefore important for controlling parasite growth and reducing parasite burden in AE.

Current clinical treatment mainly relies on benzimidazole compounds such as albendazole and mebendazole. These drugs mainly inhibit parasite growth and proliferation, but are difficult to use to eradicate infection. Long-term use is also closely associated with drug-related toxic reactions, which markedly limits their clinical application (Autier et al., 2024). Therefore, the development of new anti-echinococcal candidate drugs with well-defined mechanisms, improved safety, and greater efficacy, together with clarification of their key targets, has become an important research priority in this field.

Beyond direct parasite proliferation and tissue invasion, AE progression is also closely associated with regulation of the host immune microenvironment. T-cell subsets and their associated cytokines contribute to the balance between antiparasitic immunity, inflammation, and immune tolerance during *Echinococcus multilocularis* infection, and changes in systemic cytokine profiles have been associated with different clinical courses of AE (Grüner et al., 2022). Recent studies have also implicated the TLR4/MyD88/NF-κB signaling axis in inflammatory responses associated with *E. multilocularis* infection. Studies in AE patients and experimental mouse models have reported altered expression of TLR4/MyD88/NF-κB pathway components in association with inflammatory responses in lesion-adjacent tissues (Zhu et al., 2024). Moreover, experimental modulation of MyD88/NF-κB signaling has been reported to affect Th1/Th2 immune balance, inflammation, and hepatic fibrosis in AE models (Wang L et al., 2025). These findings provide a rationale for evaluating T-cell subsets, cytokine profiles, and TLR4/MyD88/NF-κB signaling as indicators of treatment-associated changes in the lesion immune microenvironment.

In recent years, natural products have attracted considerable attention in antiparasitic drug development. Albendazole (ABZ), which is commonly used in the clinic, can inhibit microtubulin-related processes in echinococcal larvae, block glucose uptake, and induce energy metabolism disorders, thereby inhibiting the growth of echinococcal cysts (Jing et al., 2024). This suggests that targeting energy metabolism and the cytoskeleton to limit parasite proliferation may represent an important direction for antiparasitic drug development. Isoalantolactone (ISO) is a sesquiterpene lactone derived from plants of the genus *Inula*. Studies have reported that it has multiple targets, low toxicity, and favorable pharmacological activity (Yang et al., 2024). ISO has been reported to induce intracellular reactive oxygen species (ROS) accumulation, mitochondria-associated apoptotic signaling, and changes in PI3K/AKT and NF-κB signaling in other experimental contexts (Lee et al., 2025). These reported activities provided a rationale for examining parasite-associated cellular changes and host inflammatory and immune readouts in the present study.

Multiple studies have reported insecticidal or development-inhibitory effects of ISO against several insect species, including the rice weevil (Liu et al., 2006), *Aedes albopictus* (Konishi et al., 2008), *Aedes aegypti* (Cantrell et al., 2010), and *Spodoptera litura* (Kaur et al., 2017). However, insects and cestodes are phylogenetically and physiologically distinct, and these findings do not necessarily indicate that ISO acts through similar molecular targets or metabolic pathways in *Echinococcus*. More relevantly, ISO has demonstrated *in vitro* antiprotozoal activity against *Trypanosoma brucei rhodesiense* and *Trypanosoma cruzi* (Schmidt et al., 2002), providing a more direct precedent for its potential antiparasitic activity. Although the specific molecular targets underlying the antitrypanosomal activity of ISO remain unclear, mechanistic studies of other sesquiterpene lactones in trypanosomatid parasites provide useful mechanistic context. For example, cynaropicrin has been reported to rapidly deplete intracellular glutathione (GSH) and trypanothione [T(SH)_2_] in *Trypanosoma brucei rhodesiense* through the formation of Michael-type adducts and to inhibit ornithine decarboxylase (Zimmermann et al., 2013). In *T. cruzi*, deoxymikanolide has been reported to reduce intracellular thiol levels, alter antioxidant enzyme activities, and induce mitochondrial membrane depolarization (Puente et al., 2019). These findings suggest that thiol-redox homeostasis, trypanothione-associated metabolism, and mitochondrial function may represent pharmacologically vulnerable processes for some sesquiterpene lactones in trypanosomatid parasites. However, these mechanisms have not been established specifically for ISO, and the molecular basis of its antitrypanosomal activity remains unresolved. Thus, the reported antitrypanosomal activity of ISO provides a relevant precedent for its antiparasitic potential rather than evidence of a conserved mechanism in *Echinococcus*.

In our high-throughput primary screen of 2,541 natural compounds against *Echinococcus multilocularis* protoscoleces, 40 prioritized compounds were selected for secondary validation based on preliminary activity, solubility, structural diversity, and druggability (Supplementary Figure S1). Among these candidates, ISO showed the most potent and consistent anti-protoscolex activity and was therefore selected for subsequent *in vitro* and *in vivo* evaluation.

We hypothesized that ISO would show direct anti-*E*. *multilocularis* activity and that treatment would be accompanied by measurable changes in the lesion microenvironment. Accordingly, we assessed its activity against protoscoleces, metacestode vesicles, and germinal layer cells, characterized parasite-associated mitochondrial alterations and changes in β-tubulin expression, and examined treatment-associated host lesion-microenvironment and immune readouts *in vivo*.

## 2 Methods

### 2.1 Cells and reagents

HepG2 cells, L02 human normal liver cells, and HK-2 cells were purchased from Pricella Biotechnology (Wuhan, China). HepG2 cells were cultured in Dulbecco’s modified Eagle medium (DMEM; Pricella Biotechnology) supplemented with 10% fetal bovine serum (FBS; Pricella Biotechnology) and 1% penicillin–streptomycin (PS; Pricella Biotechnology). L02 cells and HK-2 cells were cultured in minimum essential medium (MEM; Pricella Biotechnology) supplemented with 10% FBS and 1% PS. L02 cells were used as the normal human liver-cell model in the cytotoxicity assay. All cells were maintained in a humidified incubator at 37° C with 5% CO_2_ (HERAcell 150i, ThermoFisher Scientific, Waltham, MA, United States). When cell confluence reached 80%–90%, cells were detached with 0.25% trypsin-EDTA (Pricella Biotechnology). Cells were passaged at a 1:3 ratio every 2–3 days. Experiments were performed using cells between passages 3 and 6 to ensure consistent phenotypic characteristics, with the independent cell-passage batch defined as the independent experimental unit. All cell culture experiments were performed with at least 3 independent biological replicates, with 5 technical replicate wells per group in each replicate.

ISO, ABZ, and albendazole sulfoxide (ABZSO) were purchased from Yuanye Bio-Technology (Shanghai, China). ISO was dissolved in 0.01 M phosphate-buffered saline (PBS; pH 7.2; Solarbio, Beijing, China) containing 10% dimethyl sulfoxide (DMSO; Solarbio, Beijing, China) to prepare a 100 mM stock solution. ABZSO was dissolved in DMSO to prepare a 5 mg/mL stock solution. All stock solutions were protected from light and stored at −80° C. For *in vitro* assays, the final DMSO concentration was maintained at ≤0.1%, and 0.1% DMSO-treated groups were used as solvent controls. For *in vivo* administration, ISO was formulated separately as described in the animal experiments.

### 2.2 Isolation of *Echinococcus multilocularis* protoscoleces

The Qinghai isolate of *E. multilocularis* was originally derived from a naturally infected plateau pika (Ochotona curzoniae) collected in Yushu, Qinghai, China. Experimental protoscoleces used in this study were recovered from passaged Mongolian gerbils. In a Class II biosafety cabinet (ESCO Lifesciences, Changi, Singapore), the echinococcal tissues were minced and repeatedly washed with sterile saline. The suspension was filtered through four layers of sterile gauze into a 50 mL centrifuge tube. The sample was washed 8–10 times to remove host tissues. Calcareous corpuscles were removed using a 40 μm cell strainer. Intact protoscoleces were collected by sedimentation for subsequent experiments (Spiliotis et al., 2004).

Protoscolex activation was performed as follows. Protoscoleces were placed in 0.05% w/v pepsin solution (pH 2.0, prepared in DMEM) and shaken at 37° C and 125 rpm for 30 min (Fernández et al., 2002). Viability was assessed using 0.1% methylene blue staining. Only protoscolex batches with viability ≥98% were used for subsequent experiments. Sterility was confirmed by inoculating the protoscolex supernatant onto blood agar plates for 48 h. The obtained protoscoleces were used for *in vitro* culture and intraperitoneal inoculation in C57BL/6 mice. All procedures were performed with sterile consumables in a biosafety cabinet. Each independent protoscolex isolation batch recovered from passaged Mongolian gerbils was defined as one independent biological replicate, and all related *in vitro* experiments were repeated with at least three independent batches.

### 2.3 Culture of metacestode vesicles and germinal cells

Metacestode vesicle tissues were collected from the peritoneal cavity of female C57BL/6 mice infected for 2 months. The tissues were disinfected in 75% ethanol for 5 min, then minced in a biosafety cabinet and placed overnight in PBS (0.01 M, pH 7.2) containing 1% PS. After washing five times with sterile saline, vesicles were co-cultured with 5 × 10^6^ HepG2 cells in DMEM supplemented with 10% FBS and 1% PS. Cultures were maintained at 37° C with 5% CO_2_. The medium was changed twice per week. During culture, phenol red was used to identify collapsed or degenerated vesicles, which were removed. Vesicles with a diameter of 2–4 mm and intact structure were selected for experiments, and eight vesicles were used per well for all vesicle assays.

A portion of the vesicles was digested with 0.125% trypsin (Pricella Biotechnology) for 5 min to prepare germinal cells. The digestion was stopped, and cells were collected by centrifugation for subsequent experiments. The remaining vesicles were fixed in 4% paraformaldehyde for pathological identification. Each independent vesicle/germinal cell isolation batch from infected mice was defined as one independent biological replicate, and all related *in vitro* experiments were repeated with at least three independent batches.

### 2.4 Molecular identification

DNA was extracted from protoscoleces, cultured vesicles, and germinal cells using the TIANamp DNA Kit (TianGen Biotech, Beijing, China). Specific primers were used to amplify the *E. multilocularis* *nad5* and *cox1* genes and the *Echinococcus granulosus* *nad1* gene for identification. Primers were synthesized in Shanghai, and the sequences are shown in Table 1.

**TABLE 1:** Primer sequences.

Gene names	Forward (5′-3′)	Reverse (5′-3′)
*nad5*	CAT​TAA​TTA​TGG​ATG​TTT​CC	GGAAATACCCCACTATCC
*nad1*	GGT​TTT​TTA​TCG​GTA​TGT​TGG​TGT​TAG​TG	CAT​TTC​TTG​AAG​TTA​ACA​GCA​TCA​CG
*cox1*	TTG​AAT​TTG​CCA​CGT​TTG​AAT​GC	GAA​CCT​AAC​GAC​ATA​ACA​TAA​TGA

PCR was performed on a Mastercycler Nexus PCR instrument (Eppendorf, Hamburg, Germany) which was calibrated and regularly maintained. The total PCR reaction volume was 25 μL. It contained 10 μL 2 × Taq PCR Mix, 0.6 μL of each primer, 2 μL template DNA, and 6.8 μL ddH_2_O. The cycling program was as follows: 94° C for 3 min; 30 cycles of 94° C for 30 s, 61° C for 35 s, and 72° C for 30 s; and a final extension at 72° C for 5 min. PCR products were examined by 2% agarose gel electrophoresis to confirm specific amplification of the target parasite genes.

### 2.5 Cytotoxicity assay in mammalian cells

HepG2 cells, HK-2 cells, and L02 human normal liver cells were adjusted to 1 × 10^4^ cells/mL and seeded into 96-well plates (100 μL/well). Cells were pre-incubated at 37° C for 8 h, then treated with different concentrations of ISO (2.5, 5, 10, 20, 40, 80, 160, and 320 μM) for 48 h.

After treatment, the supernatant was removed. Each well received 100 μL fresh medium and 10 μL CCK-8 (Elabscience, Wuhan, China). Plates were incubated at 37° C for 1 h. Absorbance at 450 nm was measured using a microplate reader (Infinite M200, Tecan, Switzerland). Relative cell viability was calculated after subtraction of the blank wells. The 0.1% DMSO group served as the negative control. For each independent biological replicate, the absorbance values from the five technical replicate wells at each ISO concentration were averaged to obtain a single value. The corrected OD values were normalized to the corresponding 0.1% DMSO control within each biological replicate and expressed as relative OD values (%). Half-maximal inhibitory concentration (IC50) values were estimated using the AAT Bioquest online IC50 Calculator by nonlinear regression with a four-parameter logistic (4PL) model. Six independent biological replicates were performed for each concentration. Results are presented as mean ± standard deviation (SD).

### 2.6 Effects of ISO on protoscoleces

Activated protoscoleces (1,000/mL) were resuspended in serum-free RPMI 1640 medium (Procell, Wuhan) and seeded into 6-well plates (3 mL per well). Equal volumes of ISO working solutions were added to achieve final ISO concentrations of 2.5, 5, 10, 20, 40, 80, 160, and 320 μM. The negative control was 0.1% DMSO. The positive control was ABZSO (40 μM), selected based on preliminary optimization showing reproducible anti-protoscolex activity over the 7-day assay window; this condition was used as an assay benchmark rather than as an equimolar comparator for ISO. Protoscoleces were cultured continuously for 7 days at 37° C with 5% CO_2_.

Each day, 0.5 mL of suspension was collected. After removing the supernatant, samples were allowed to sediment naturally. Ten microliters of protoscoleces were mixed with an equal volume of 0.1% eosin solution and incubated for 3 min. Under a BX53 inverted microscope (Olympus, Tokyo, Japan), 100 protoscoleces were randomly counted. Dead protoscoleces were stained red, whereas live protoscoleces were unstained. Survival numbers were recorded. Each group had 3 technical replicate wells in each experiment, and the experiment was independently repeated 3 times using protoscoleces from 3 independent isolation batches. The independent experimental unit was an individual protoscolex isolation batch. Some samples were fixed in 2.5% glutaraldehyde for SEM and TEM.

### 2.7 Scanning electron microscopy and transmission electron microscopy

Vesicles or protoscoleces were fixed in 2.5% glutaraldehyde (0.01 M PBS, pH 7.2) at 4° C overnight. Samples were washed three times with PBS (15 min each) and then fixed in 1% osmium tetroxide at room temperature for 2 h.

For SEM, samples were dehydrated through graded ethanol (30%, 50%, 70%, 80%, 90%, 95%, and 100%; 15 min each). Samples were treated with hexamethyldisilazane (Sigma-Aldrich, St. Louis, MO, United States) for 10 min and air-dried. Dried samples were sputter-coated with gold-palladium to 10 nm (E-1045, HITACHI, Tokyo, Japan). Imaging was performed on a JSM-IT700HR scanning electron microscope (JEOL, Tokyo, Japan) at an accelerating voltage of 20 kV. Each group was independently repeated three times.

For TEM, samples were dehydrated through graded acetone (30%, 50%, 70%, 80%, 90%, 95%, and 100%; 100% acetone was replaced three times). Samples were infiltrated sequentially with acetone:Epon 812 resin (1:1) for 2 h and (1:3) for 4 h, and then embedded. Polymerization was performed at 60° C for 48 h. Ultrathin sections (50 nm) were prepared using a Leica UC7 ultramicrotome and collected on 200-mesh copper grids. Sections were stained with 2% uranyl acetate for 15 min and lead citrate for 10 min. Sections were observed using a JEM-1400FLASH transmission electron microscope (JEOL) at 80 kV. Each group was independently repeated three times.

Each SEM and TEM experiment was independently repeated 3 times, with samples from 3 different mice or 3p independent parasite isolation batches per group. The independent experimental unit was a single mouse or an independent parasite isolation batch.

### 2.8 Effects of ISO on germinal cells

Germinal cells obtained by digesting vesicle tissues with 0.125% trypsin were seeded into black 384-well plates (approximately 2 × 10^4^ cells per well). ISO at different concentrations (0.5–320 μM) was added. The final volume was 50 μL. The 0.1% DMSO group served as the vehicle control. After incubation at 37° C with 5% CO_2_ for 5 days, 25 μL CellTiter-Glo solution and 1% Triton X-100 were added to each well. Plates were incubated at room temperature in the dark for 15 min. Signals were measured using a luminescence microplate reader (Infinite F200, Tecan).

For each independent biological replicate, the luminescence values from the three technical replicate wells at each ISO concentration were averaged to obtain a single value. Relative viability was normalized to the corresponding 0 μM control group within the same biological replicate. IC50 values were estimated using the AAT Bioquest IC50 Calculator (https://www.aatbio.com/tools/ic50-calculator) by nonlinear regression with a four-parameter logistic (4PL) model. Four independent biological replicates were performed for each concentration, with each independent germinal cell isolation batch defined as one biological replicate. Results are presented as mean ± SD.

### 2.9 PGI release assay for metacestode vesicles

PGI release from metacestode vesicles after ISO treatment was evaluated using a phosphoglucose isomerase (PGI) release assay. Metacestode vesicles were purified with 2% sucrose, and vesicles measuring approximately 2–4 mm in diameter were selected and washed several times with PBS. The purified vesicles were maintained in phenol red-free DMEM supplemented with 1% penicillin/streptomycin and distributed into 48-well culture plates. ISO was added at final concentrations of 5, 10, 20, 40, 80, 160, and 320 μM. Vesicles treated with 0.1% DMSO served as the vehicle control (0 μM ISO), whereas 0.1% Triton X-100 was used as the positive control representing maximal PGI release. The vesicles were incubated at 37° C in a humidified atmosphere containing 5% CO_2_ for 5 days. Culture supernatants were then collected and stored at −80° C until analysis. PGI activity in the supernatants was quantified using a commercial PGI assay kit, and absorbance was measured at 340 nm using an Infinite M200 microplate reader (Tecan) (Liu et al., 2021b; Rufener et al., 2018). PGI release was calculated as follows: PGI release (%) = [(OD_ISO_ − OD_blank_)/OD_Triton X-100_] × 100%.

The half-maximal effective concentration (EC_50_) was calculated from the ISO concentration–response data using an online EC_50_ calculator. Three independent experiments were performed, and the results are presented as mean ± SD.

### 2.10 *In vivo* experiments

For this study, experimental protoscoleces used in the *in vitro* assays were recovered from passaged Mongolian gerbils, whereas female C57BL/6 mice were used for the *in vivo* efficacy and toxicity experiments. The Qinghai isolate was originally derived from a naturally infected plateau pika. All animals were housed under specific pathogen-free (SPF) conditions at 22° C ± 2° C with 55% ± 5% relative humidity and a 12 h light/dark cycle. Ventilated cages were used, with five animals housed per cage. Animals had free access to standard sterile chow and water. The experimental protocol was approved by the Ethics Committee of the Affiliated Hospital of Qinghai University (NO. P-SL-2024-001). The study followed the ARRIVE guidelines and the General Requirements for Laboratory Biosafety (GB19489-2008). Anesthesia was performed using Zoletil 50 (50 mg/kg) + xylazine (13.75 mg/kg). When endpoint signs occurred, including dyspnea, refusal to eat, or inability to stand, animals were euthanized by cervical dislocation.

Efficacy experiment: Female C57BL/6 mice were intraperitoneally injected with 200 μL *E. multilocularis* vesicle suspension to establish the AE infection model. At 6 weeks post-infection, mice were randomized using a computer-generated random sequence into four groups (n = 8 per group): vehicle control, ABZ positive control (100 mg/kg), ISO low-dose (10 mg/kg), and ISO high-dose (40 mg/kg) groups. For *in vivo* administration, ISO was formulated in normal saline containing 1% (v/v) DMSO at the required dosing concentrations. Vehicle-control mice received an equal volume of the same vehicle without ISO. All treatments were administered by gavage once daily at a fixed time (0.3 mL per mouse) for six consecutive weeks. At the end of the 6-week treatment period (12 weeks post-infection), orbital blood was collected before mice were euthanized by cervical dislocation. Metacestode tissues, spleen, liver, kidney, and perilesional host tissues were collected for subsequent experiments, and lesion weight and spleen index were calculated. Spleen index = [spleen weight (mg)/(mouse body weight − metacestodes weight) (g)] (Zhang et al., 2018). Metacestode tissues were fixed in 2.5% glutaraldehyde for TEM observation, and the remaining collected tissues were stored at −80° C for subsequent analyses. To reduce assessment bias, all outcome evaluations were completed under blinded conditions by an investigator unaware of the group allocation.

### 2.11 *In vivo* toxicity experiment

Toxicity experiment: Twelve female C57BL/6 mice (6–8 weeks old) were randomly assigned to a vehicle-control group and an ISO 40 mg/kg group (n = 6 per group), with each individual mouse defined as one biological replicate. The vehicle-control group received 0.3 mL of the matched vehicle (normal saline containing 1% [v/v] DMSO) by gavage daily, whereas the ISO group received ISO at 40 mg/kg formulated in the same vehicle (0.3 mL per mouse) by gavage daily for six consecutive weeks. After the last administration, orbital blood was collected for hematological and biochemical analyses. Blood cell analysis was performed using a BC-5000Vet analyzer (Mindray, Shenzhen, China). For serum biochemistry, blood samples were allowed to clot at room temperature for 1 h and then centrifuged at 1,500 *g* for 15 min at 4° C. Serum alanine aminotransferase, aspartate aminotransferase, total bilirubin, direct bilirubin, indirect bilirubin, total protein, albumin, alkaline phosphatase, creatinine, and blood urea nitrogen were measured using an automated biochemical analyzer (Chemray 800, Rayto, Shenzhen, China) according to the manufacturer’s instructions. Mice were then euthanized by cervical dislocation, and liver and kidney tissues were fixed in 4% paraformaldehyde for 3 days for routine paraffin embedding, sectioning (4 μm), and H&E staining. All outcome measurements were completed under blinded conditions by an investigator unaware of the group allocation.

### 2.12 Flow cytometry

Perilesional host tissues and peripheral blood were collected from eight mice per group. CD4^+^ and CD8^+^ T-cell proportions were analyzed in both perilesional tissues and peripheral blood, whereas Th1, Th2, Th17, and Treg subsets were analyzed in perilesional tissues. Perilesional host tissue samples were minced and digested with 1 mg/mL collagenase II and 100 U/mL DNase I in RPMI 1640 medium at 37° C for 45 min with gentle shaking to prepare single-cell suspensions. The suspension was filtered through a 70 μm cell strainer, centrifuged at 500 *g* for 5 min at 4° C, and resuspended in PBS. The cell suspension was layered onto Ficoll-Paque density gradient medium and centrifuged at 800 *g* for 20 min at room temperature with no brake. The lymphocyte layer was collected, washed twice with pre-cooled PBS, and resuspended in flow-cytometry staining buffer for subsequent staining.

Surface staining was performed as follows. A total of 1 × 10^6^ live cells per sample were stained with CD3ε-V450, CD4-APC, and CD8a-PE antibodies and incubated at 4° C for 30 min in the dark. After washing twice with staining buffer, samples were analyzed using a NovoCyte flow cytometer (ACEA Biosciences).

For intracellular cytokine staining, cells were stimulated with 50 ng/mL phorbol 12-myristate 13-acetate (PMA), 1 μg/mL ionomycin, and brefeldin A (BFA)/Monensin protein transport inhibitor cocktail for 6 h at 37° C with 5% CO_2_. Cells were then stained with surface markers (CD3ε-redFluor 710 and CD4-APC-Cy7) at 4° C for 30 min, followed by fixation and permeabilization using a commercial fixation/permeabilization kit (eBioscience) according to the manufacturer’s instructions. Cells were then stained with cytokine antibodies (IFN-γ-PE for Th1 cells, IL-4-APC for Th2 cells, and IL-17A-BL421 for Th17 cells) for 30 min at 4° C in the dark.

For Treg staining, cells were stained with anti-CD4-APC-Cy7 and CD25-APC at 4° C for 30 min, then fixed and permeabilized using a Foxp3/Transcription Factor Staining Buffer Kit (eBioscience). Cells were incubated with Foxp3-PE or the corresponding isotype control antibody at 4° C for 45 min in the dark.

For flow-cytometric analyses, perilesional tissues and peripheral blood were collected from 8 mice per group, and each mouse was treated as one independent biological replicate. Repeated assay runs were performed only to verify technical stability and were not treated as additional biological replicates.

### 2.13 Enzyme-linked immunosorbent assay (ELISA) analysis

ELISA kits were used to measure the levels of IL-4, IL-17, IL-6, IL-10, and IFN-γ in mouse serum, with eight mice per group included for analysis (n = 8 per group). Blood was collected and allowed to clot at room temperature for 1 h. Serum was obtained by centrifugation at 4° C and 1,500 × g for 15 min. All reagents and samples were equilibrated to room temperature. Standards were serially diluted according to the kit instructions to generate a standard curve. Samples were diluted based on pre-experiment results. A total of 100 μL of standards, samples, or blanks (sample diluent) was added to each well of the ELISA plate and incubated at 37° C for 90 min. Wells were washed three times. Then 100 μL horseradish peroxidase (HRP) conjugate was added and incubated at 37° C for 30 min. After washing five times with TBST, 3,3′,5,5′-tetramethylbenzidine (TMB) substrate was added and incubated at 37° C in a constant-temperature dry incubator. When obvious graded blue color appeared in the standard wells, 50 μL stop solution was added. Absorbance at 450 nm was measured using a microplate reader. Concentrations were calculated from the standard curve. For ELISA analysis, serum samples from eight mice per group were analyzed, with each mouse serving as one independent biological replicate. Technical duplicates were used for assay quality control, and repeated assay runs were performed only to verify technical stability.

### 2.14 Histological staining

Metacestode tissues from the efficacy experiment were fixed in 4% paraformaldehyde for 48 h. Liver and kidney tissues from the toxicity experiment were fixed in 4% paraformaldehyde for 3 days. After dehydration through graded ethanol, tissues were routinely embedded in paraffin and sectioned at 4 μm. Three tissue sections per mouse were examined, with five random fields imaged per section. All morphological analyses were performed by an investigator blinded to group allocation. The individual mouse was considered the independent experimental unit.

### 2.15 H&E staining

Sections were stained with 0.5% hematoxylin (Servicebio, Wuhan, China) for 5 min, rinsed with running tap water, differentiated with 1% acid alcohol for 3 s, and blued with 0.2% ammonia water for 30 s. Sections were then counterstained with 0.5% eosin for 1 min. Sections were dehydrated with 95% ethanol twice (2 min each) and 100% ethanol (2 min), mounted with neutral gum and coverslips, and observed using a BX53 microscope (Olympus) with a FL-20 digital camera (XingtuSun company, Shenzhen, China). Images were acquired at ×200 total magnification.

### 2.16 Periodic acid–Schiff (PAS) staining

Paraffin sections were dewaxed in xylene and rehydrated through graded ethanol to distilled water. Sections were oxidized with 0.5% periodic acid for 6 min, rinsed with running water and then with distilled water, and stained with Schiff reagent for 15 min. After rinsing with running water for 10 min, sections were counterstained with 0.5% hematoxylin for 1 min. Acid differentiation solution was applied for 3 s, followed by rinsing with running water for 10 min. Sections were dehydrated in 95% ethanol for 3 s, followed by two changes of absolute ethanol (5 s each), cleared twice in xylene (2 min each), and mounted with neutral resin. Images were acquired at 200× using a BX53 microscope (Olympus). Five random fields were selected for each sample. Positive reactions appeared red or reddish-purple.

### 2.17 JC-1 staining for mitochondrial membrane potential

The JC-1 working solution was prepared according to the instructions of the JC-1 Mitochondrial Membrane Potential Assay Kit (Beyotime Biotechnology, Shanghai, China). E. multilocularis protoscoleces were treated with 10 μM ISO for 3 days. After removal of the culture medium, the protoscoleces were washed twice with PBS and incubated with 2 mL JC-1 working solution at 37° C for 20 min. After staining, the protoscoleces were washed twice with JC-1 staining buffer, resuspended in 1 mL DMEM, and immediately observed and imaged under a fluorescence microscope.

### 2.18 Real-time quantitative PCR(RT-qPCR)

Parasite tissues used for E. multilocularis gene-expression analysis were snap-frozen in liquid nitrogen. Total RNA was extracted using TRIzol (ThermoFisher Scientific) according to the manufacturer’s instructions. Genomic DNA was removed with DNase I (1 U/μL, 30 min, 37° C; ThermoFisher Scientific). RNA purity was assessed using NanoDrop 2000 (ThermoFisher Scientific). Acceptable purity criteria were A260/A280 = 1.8–2.0 and A260/A230 = 2.0–2.2. Samples that did not meet the criteria were re-extracted. RNA integrity was checked by 1.5% agarose gel electrophoresis. Reverse transcription was performed using 2 μg RNA and the FastKing RT SuperMix Kit (TianGen Biotech). Real-time PCR was performed using the SuperReal PreMix Plus (SYBR Green) Kit (TianGen Biotech) and an ABI QuantStudio 5 instrument (Applied Biosystems, Carlsbad, CA, United States).

Primer sequences used for RT-qPCR are provided in Table 2. The reaction volume was 20 μL and contained 10 μL SuperReal PreMix Plus (2×), 0.6 μL forward primer (10 μM), 0.6 μL reverse primer (10 μM), 0.4 μL ROX (50×), 2 μL cDNA, and 6.4 μL H_2_O. Cycling conditions were 95° C for 15 min, followed by 40 cycles of 95° C for 15 s and 60° C for 30 s. Melting-curve analysis was performed to confirm amplification specificity, with 95° C for 15 s, 60° C for 1 min, a gradual increase from 60° C to 95° C at 0.3° C/step, and 95° C for 15 s. For relative quantification of parasite β-tubulin transcripts, E. multilocularis actin (Em actin) was used as the reference gene. Relative expression was calculated using the 2^−ΔΔCT^ method (Livak and Schmittgen, 2001). Each sample was analyzed in technical triplicate. Three independent protoscolex isolation batches were analyzed as biological replicates.

**TABLE 2:** Primer sequences used for RT-qPCR.

Gene	Species/target	Forward (5′-3′)	Reverse (5′-3′)
Actin	*Echinococcus multilocularis*/reference gene	CGA​TGT​GGG​CTC​CGG​TCT​CTG	GTT​GGG​CCG​CAT​GAC​CAA​CA
β-tubulin	*Echinococcus multilocularis*/target gene	TGC​CTG​GTT​TTG​CCC​CGC​TT	AGG​ATC​GCA​AGC​CGC​CAT​CA

### 2.19 Western blot

For analysis of parasite β-tubulin, protoscoleces from untreated and ISO-treated groups were collected, washed with PBS, lysed in RIPA buffer containing protease inhibitors, and processed for Western blotting as described below. Mouse perilesional host tissues were homogenized on ice in RIPA lysis buffer (1:10, w/v; 25 mM Tris-HCl pH 7.6, 150 mM NaCl, 1% NP-40, 1% sodium deoxycholate, 0.1% SDS; ThermoFisher Scientific) plus protease/phosphatase inhibitors (1:100, Sangon Biotech, Shanghai, China). Samples were lysed by low-power probe sonication (H150, OuHor, Shanghai, China) at 20% amplitude for 3 cycles of 3 s on/9 s off. Samples were centrifuged at 12,000 × g for 15 min at 4° C, and the supernatant was collected. The supernatant was diluted with 5× protein loading buffer and boiled at 95° C for 10 min, after which the protein samples were aliquoted and stored at −80° C. Protein concentration was determined by bicinchoninic acid (BCA) assay (ThermoFisher Scientific).

A total of 30 μg protein per sample was separated by 8%–12% sodium dodecyl sulfate–polyacrylamide gel electrophoresis (SDS-PAGE; gradient gel according to protein molecular weight) and transferred to a 0.2 μm polyvinylidene difluoride (PVDF) membrane (Merck Millipore, Darmstadt, Germany). Membranes were blocked for 1 h in Tris-buffered saline containing Tween-20 (TBST; 10 mM Tris, 150 mM NaCl, 0.05% Tween-20, pH 7.2) containing 5% bovine serum albumin (BSA) for phosphorylated proteins or non-fat milk for non-phosphorylated proteins. For parasite β-tubulin detection, membranes were incubated with a β-tubulin primary antibody (1:1,000, Cell Signaling Technology, Danvers, MA, United States; Cat. No. 2146) at 4° C overnight. For host-tissue analyses, membranes were incubated with the corresponding primary antibodies at 4° C overnight, with detailed antibody information listed in Table 3. The next day, membranes were washed with TBST (3 times, 10 min each). Membranes were incubated with HRP-conjugated secondary antibody (goat anti-rabbit IgG or goat anti-mouse IgG, 1:5,000, Proteintech) at room temperature for 1 h. Signals were developed using enhanced chemiluminescence (ECL) reagent (ThermoFisher Scientific) and detected using a SageCreation imaging system (SageCreation, Beijing, China). Band intensities were quantified using ImageJ 1.51j8 software (National Institutes of Health, Bethesda, MD, United States). After background subtraction, non-phosphorylated host proteins were normalized to β-actin, p-Smad2/3 was normalized to total Smad2/3, p-NF-κB p65 was normalized to total NF-κB p65, and parasite β-tubulin was normalized to parasite actin. For parasite β-tubulin Western blot analysis, three independent protoscolex isolation batches were analyzed, with each isolation batch considered one biological replicate. For Western blot analyses of mouse perilesional host tissues, six mice per group were included as independent biological replicates (n = 6 mice per group, 1 mouse per sample). Each mouse was considered one independent experimental unit.

**TABLE 3:** Primary antibodies used for Western blotting and immunohistochemistry analyses.

Antibody target	Manufacturer	Catalog No.	Western blot dilution	Immunohistochemistry dilution
Bax	Proteintech, Wuhan, China	50599-2-Ig	1:1,000	—
Bcl-2	Proteintech, Wuhan, China	26593-1-AP	1:1,000	—
Cleaved caspase-3	Cell signaling Technology, Danvers, MA, United States	11872	1:1,000	1:300
Col1a1	Proteintech, Wuhan, China	66761-1-Ig	1:1,000	—
Col3a1	Proteintech, Wuhan, China	22734-1-AP	1:1,000	—
MMP2	ABclonal, Wuhan, China	A19080	1:1,000	—
MMP9	ABclonal, Wuhan, China	A24220	1:1,000	—
PCNA	ABclonal, Wuhan, China	A12427	1:1,500	1:500
CD31	ABclonal, Wuhan, China	A19014	1:800	1:400
RUNX2	ABclonal, Wuhan, China	A2851SP	1:1,000	—
SPP1 (Osteopontin)	Proteintech, Wuhan, China	30200-1-AP	1:1,000	—
TGF-β1	ABclonal, Wuhan, China	A25313	1:1,000	1:300
Smad2/3	Beyotime, Shanghai, China	AF8001	1:1,200	—
p-Smad2/3 (Ser465/467)	Beyotime, Shanghai, China	AF5920	1:1,200	—
TLR4	Proteintech, Wuhan, China	19811-1-AP	1:1,000	—
MyD88	Proteintech, Wuhan, China	67969-1-Ig	1:1,000	—
NF-κB p65	Proteintech, Wuhan, China	33259-1-AP	1:1,000	—
β-Tubulin	Cell signaling Technology, Danvers, MA, United States	2146	1:1,000	—
p-NF-κB p65 (Ser536)	Proteintech, Wuhan, China	80379-2-RR	1:800	1:300
β-actin	ABclonal, Wuhan, China	AC026	1:2000	—

### 2.20 Molecular docking

Target proteins included *Echinococcus multilocularis* β-tubulin-I (Q8M1H6) and β-tubulin-III (Q8M1H8). Protein sequences were obtained from the UniProt database (https://www.uniprot.org/). Targets also included an *Echinococcus* gluconeogenesis enzyme (A0A068YEW5) and a glycolysis enzyme (Q9NFZ5). Predicted 3D protein structures were obtained from the AlphaFold Protein Structure Database. The small-molecule structure of ISO (SDF) was downloaded from the PubChem database (CID: 73285), energy-minimized using Chem3D, and converted to PDBQT format for docking. Protein structures were prepared for docking using PyMOL and AutoDockTools (v1.5.6). Polar hydrogens and Gasteiger charges were added, and the prepared structures were saved in PDBQT format.

Docking was performed using AutoDock Vina (v1.2.0). For each target protein, a grid box of X = 40, Y = 40, and Z = 40 was defined to encompass the putative binding pocket used for docking. After docking, the top-ranked docking pose was selected based on the predicted binding affinity (Vina score). PyMOL was used to visualize key hydrogen bonds and hydrophobic interactions. Each target protein was docked three times to verify the consistency of the docking results. Residues located within 4 Å of the docked ISO molecule were identified as putative interacting residues.

### 2.21 Statistical analysis

Data were analyzed using GraphPad Prism 8.2.1 (GraphPad Software, San Diego, CA, United States). Normality was tested using the Shapiro–Wilk test, and homogeneity of variance was tested using Levene’s test. When data were normally distributed with equal variances, an unpaired two-tailed Student’s t-test was used for two-group comparisons, and one-way ANOVA followed by Tukey’s multiple-comparisons test was used for multi-group comparisons. For non-normally distributed data, the Mann–Whitney U test was used for two-group comparisons, whereas the Kruskal–Wallis test followed by Dunn’s multiple-comparisons test was used for multi-group comparisons. For longitudinal body-weight data and time-course protoscolex survival data, repeated-measures analyses were performed using a mixed-effects model, or repeated-measures ANOVA when assumptions were met, followed by appropriate *post hoc* multiple-comparison correction. For *in vitro* assays, the independent experimental unit was an independent parasite or cell-preparation batch; for *in vivo* experiments, the independent experimental unit was an individual mouse. Technical replicate wells, microscopic fields, counted protoscoleces, and repeated assay runs were used only to generate a summary value for each independent experimental unit and were not treated as independent biological replicates. Statistical analyses were performed using these summary values to avoid pseudoreplication. Results are presented as mean ± SD for normally distributed data or median (interquartile range [IQR]) for non-normally distributed data. Sample size for the *in vivo* experiments was estimated by pre-experimental power analysis with a significance level of α = 0.05 and a target power of 1−β = 0.80, based on pilot data from lesion-burden measurements obtained in preliminary experiments. A *p* value <0.05 was considered statistically significant.

## 3 Results

### 3.1 Establishment of *in vitro E. multilocularis* metacestode models and anti-parasitic activity of ISO

We successfully isolated and cultured *Echinococcus multilocularis* metacestode vesicles and germinal layer cells *in vitro* and confirmed their origin by morphological and molecular identification. Based on this model, ISO increased PGI release from metacestode vesicles and reduced germinal layer cell viability (Figure 1).

**FIGURE 1:**
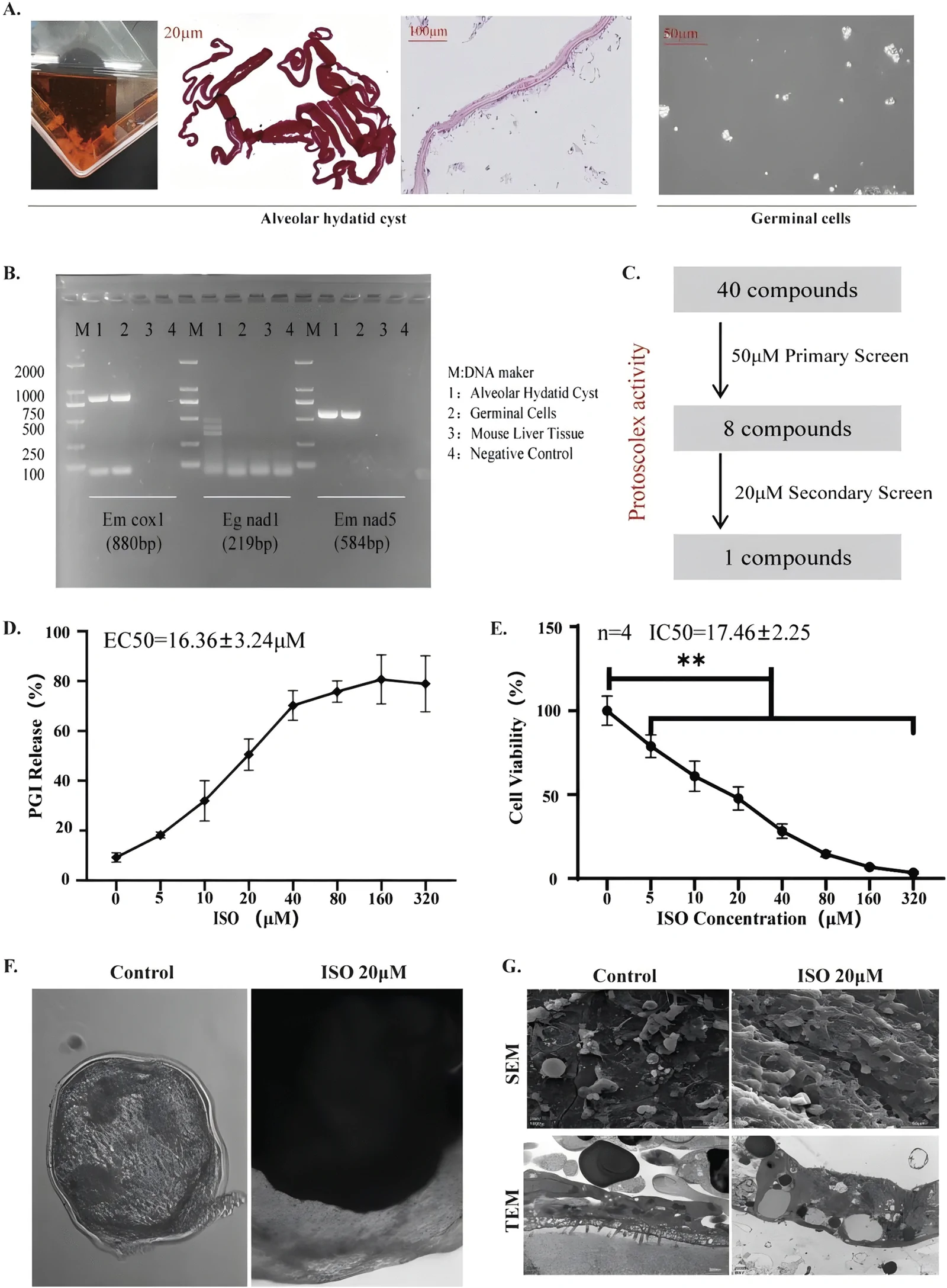
Establishment of an *in vitro* culture system of *E. multilocularis* and *in vitro* pharmacodynamic characterization of ISO. **(A)** *In vitro* culture of *E. multilocularis* vesicles (left 1), histological staining (PAS staining, left 2; H&E staining, left 3), and microscopic observation of germinal layer cells (right 1). **(B)** PCR identification of *E. multilocularis* (Em) and *E. granulosus* (E.g.,) specific genes (M: DNA marker; 1: *E. multilocularis* vesicles; 2: germinal layer cells; 3: mouse liver tissue; 4: negative control). **(C)** Screening workflow for active anti-protoscolex compounds. ISO was finally selected. **(D)** Dose-response curve of ISO-induced PGI release from vesicles and the half-maximal effective concentration (EC50). **(E)** Inhibition curve of ISO on germinal layer cell viability and the half-maximal inhibitory concentration (IC50). **(F)** Morphological differences of vesicles between the control group and the 20 μM ISO-treated group under light microscopy. The control group showed intact vesicle structure. In the 20 μM ISO group, the laminated layer separated from the germinal layer, and the germinal layer shrank. **(G)** Ultrastructural changes of germinal layer cells between the control group and the 20 μM ISO-treated group under SEM and TEM. Under SEM, many microvesicles were attached to the inner surface of vesicles in the control group. Under TEM, the vesicle wall was divided into three layers, including the laminated layer, syncytial layer, and germinal layer. Many microvilli protruded into the laminated layer. Germinal layer cells consisted of connective tissue, muscle cells, glycogen-containing storage cells, and undifferentiated cells. Many mitochondria were observed. In the 20 μM ISO group, no obvious vesicle budding was observed on the inner surface. The laminated layer and germinal cell layer were separated. Marked structural and degenerative alterations were observed in germinal layer cells.

Macroscopically, isolated vesicle tissues and many newly budded small vesicles were observed in the culture flask. The vesicles grew in suspension and were approximately 2–4 mm in diameter. Most vesicles were round or oval and contained transparent cyst fluid. By H&E staining, a distinct cellular layer was observed on the inner surface of the vesicles, corresponding to the germinal layer. Outside the germinal layer, a light-pink acellular lamellar structure was observed, corresponding to the laminated layer. The laminated layer of *E. multilocularis* vesicles is a cuticular structure rich in carbohydrate-protein complexes. By PAS staining, a clear deep-red glycan-rich structure was observed. After 7 days of continuous culture, germinal layer cells showed a proliferative trend and aggregated growth (Figure 1A). PCR showed that Em *cox1* (880 bp) and Em *nad5* (584 bp) bands were amplified in vesicles and germinal layer cells. No specific bands were detected in mouse liver tissues or in the negative control. The expected parasite-specific PCR products confirmed the E. multilocularis origin of the vesicles and germinal layer cells (Figure 1B).

Forty prioritized compounds from the primary screen of 2,541 natural compounds were subjected to secondary evaluation. In the first validation round, 40 candidate compounds were tested at 50 μM for 3 days, and eight compounds showed complete protoscolex-killing activity (Supplementary Figure S1). In the second round, these active compounds were retested at 20 μM for 3 days, and ISO remained among the most potent compounds, supporting its selection for subsequent mechanistic and *in vivo* evaluation (Figure 1C; Supplementary Figure S2). A phosphoglucose isomerase (PGI) release assay was used to evaluate vesicle damage. ISO increased PGI release in a dose-dependent manner, with an EC50 of 16.36 ± 3.24 μM, indicating progressive loss of vesicle integrity (Figure 1D). In parallel, ISO reduced the viability of germinal layer cells in a concentration-dependent manner, with an IC50 of 17.46 ± 2.25 μM (Figure 1E). Further morphological analysis showed that 20 μM ISO caused separation of the laminated layer from the germinal layer and marked shrinkage of the germinal layer under light microscopy, SEM, and TEM (Figures 1F,G). SEM further showed loss of the normal budding microvesicles on the inner surface of the vesicles, whereas TEM revealed separation of the laminated and germinal layers and marked structural alterations in germinal layer cells. The morphological and ultrastructural changes were consistent with the loss of vesicle integrity indicated by the PGI and germinal-cell viability assays.

### 3.2 ISO directly kills protoscoleces *in vitro* and is associated with mitochondrial perturbation and structural alterations

Protoscolex survival analysis showed that ISO had clear dose- and time-dependent parasiticidal activity. Compared with the 40 μM ABZSO positive-control group, ISO showed faster loss of protoscolex viability at concentrations of 20 μM and above (Figure 2A). Under 10 μM ISO or 40 μM ABZSO, protoscoleces required about 5 days to die completely. Under 20 μM ISO, about 3 days were required. At concentrations ≥40 μM, all protoscoleces died within 1 day. Under light microscopy, after treatment with 10 μM ISO for 3 days, protoscoleces showed morphological changes including surface contraction, irregular contours, deeper cytoplasmic staining, and increased fragmentation (Figure 2B). With increasing dose, the survival rate of protoscoleces decreased in all ISO-treated groups. These morphological alterations were consistent with the reduced proportion of viable protoscoleces following ISO exposure. Further microscopy showed that the compound induced changes ranging from gross morphology to subcellular structures, including mitochondria (Figures 2C,D). Molecular docking predicted potential interactions of ISO with A0A068YEW5 and Q9NFZ5 (Figure 2E). After ISO treatment, β-tubulin mRNA and protein levels in protoscoleces were downregulated (Figure 2F). JC-1 staining (Figure 2D) revealed that protoscoleces in the untreated control group exhibited strong red aggregated JC-1 fluorescence, consistent with relatively preserved mitochondrial membrane potential. In contrast, protoscoleces treated with 10 μM ISO showed a marked reduction in red aggregated fluorescence and a prominent increase in green monomer fluorescence. Quantitative analysis showed that 10 μM ISO treatment resulted in a significant decrease in the JC-1 red/green fluorescence ratio compared with the untreated control group (*p* < 0.01), consistent with mitochondrial membrane potential depolarization. The JC-1, ultrastructural, and β-tubulin data showed that ISO exposure was associated with mitochondrial membrane potential depolarization, structural injury, and reduced β-tubulin expression in protoscoleces.

**FIGURE 2:**
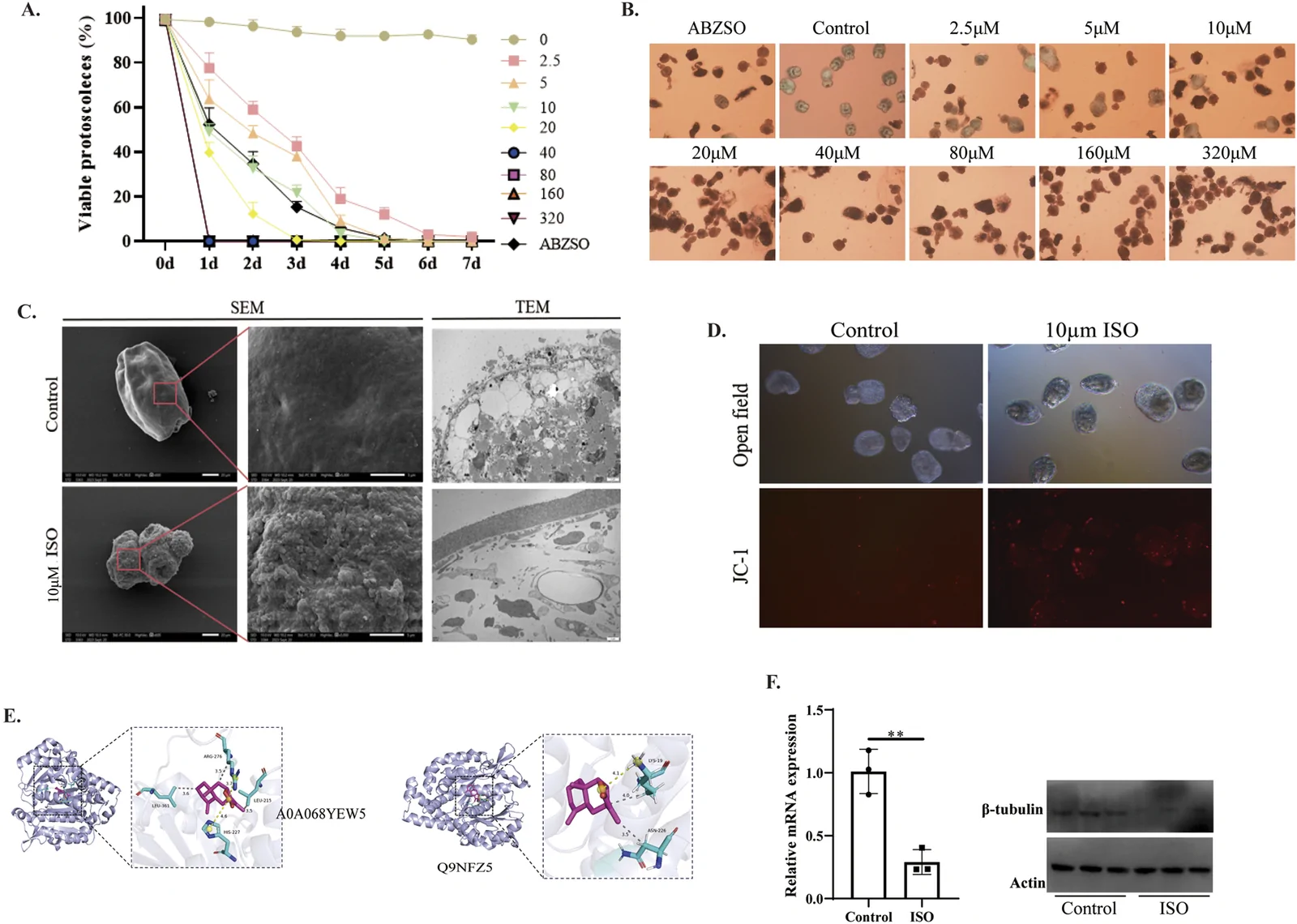
Effects of ISO on *E. multilocularis* protoscoleces. **(A)** Time-course changes in protoscolex survival after ABZSO and different concentrations of ISO. **(B)** Morphological differences of protoscoleces under light microscopy after ABZSO and different concentrations of ISO. **(C)** Ultrastructural changes of protoscoleces between the control group and the 10 μM ISO-treated group under SEM and TEM. Under SEM, hooks were lost, the surface shrank, and surface microvilli detached and disappeared after 10 μM ISO. Under TEM, the characteristic vesicular structures were markedly absent after 10 μM ISO, indicating that ISO damaged the internal structure of vesicular bodies. **(D)** Differences in mitochondrial membrane potential of *E. multilocularis* protoscoleces between the untreated control group and the 10 μM ISO-treated group observed under bright field and JC-1 staining. Protoscoleces in the 10 μM ISO-treated group showed a significant reduction in JC-1 red aggregated fluorescence, a marked increase in green monomer fluorescence, and a decreased red/green fluorescence ratio, consistent with mitochondrial membrane potential depolarization. **(E)** Molecular docking models of ISO with *E. multilocularis* proteins A0A068YEW5 and Q9NFZ5 (showing key hydrogen bonds/hydrophobic interactions and binding-site residues). **(F)** β-tubulin mRNA and protein expression levels in protoscoleces in the control group and the ISO-treated group.

### 3.3 Evaluation of ISO cytotoxicity and *in vivo* therapeutic efficacy against AE

To evaluate the *in vitro* and *in vivo* effects of ISO, we first assessed its cytotoxicity in different human-derived cells. ISO did not significantly reduce the viability of L02 human normal liver cells at concentrations up to 160 μM. At 320 μM, ISO significantly reduced the viability of L02, HK-2, and HepG2 cells (*p* < 0.01) (Figure 3A).

**FIGURE 3:**
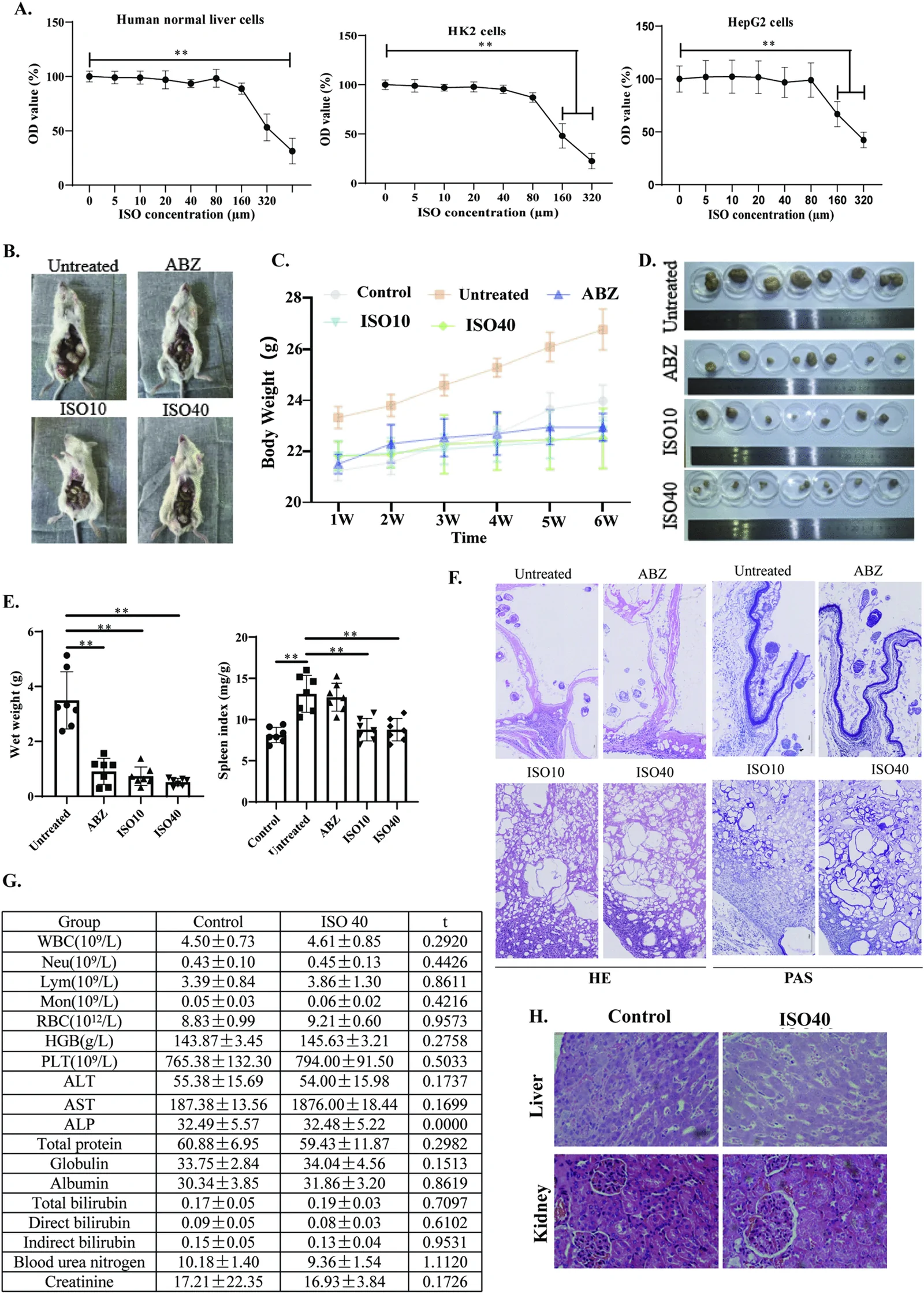
*In vitro* and *in vivo* safety of ISO and its anti-AE efficacy. **(A)** Differences in ISO cytotoxicity in L02 human normal liver cells, HK-2 cells, and HepG2 cells. **(B)** Gross anatomy of mice in different treatment groups. **(C)** Body weight trends of mice in each group over the 6-week treatment period. **(D)** Gross appearance of hepatic alveolar echinococcosis lesions in each group. **(E)** Comparison of lesion burden and spleen index among groups. **(F)** H&E and PAS staining of lesion tissues in each group. H&E staining was used for morphological assessment of lesion pathology, whereas PAS staining was used to visualize parasite-associated structures. **(G)** Toxicity assay in uninfected mice treated with the matched vehicle or ISO (40 mg/kg). **(H)** H&E staining of liver and kidney tissues from uninfected mice in the toxicity assay.* *Indicates p* < 0.05. ** *Indicates p* < 0.01.

In the animal efficacy experiment, ABZ (100 mg/kg), ISO 10 mg/kg, and ISO 40 mg/kg reduced lesion progression over the 6-week treatment period, without obvious body-weight loss (Figures 3B,C). These treatments also inhibited progression of liver lesions (*p* < 0.01) (Figures 3D,E). In addition, ISO 10 mg/kg and ISO 40 mg/kg reduced the spleen index (*p* < 0.01) (Figure 3E). Histological examination by H&E staining revealed morphological changes consistent with tissue degeneration and necrosis in lesions from the ISO-treated groups (Figure 3F). Hematological and biochemical analyses in the separate animal toxicity experiment showed no significant differences in the measured parameters between the ISO 40 mg/kg group and the control group (Figure 3G). Histological examination of liver and kidney tissues showed generally preserved tissue architecture in both the control and ISO 40 mg/kg groups. Hepatic morphology and renal glomerular and tubular structures were broadly comparable between the two groups, with no obvious treatment-related histopathological abnormalities observed (Figure 3H). Overall, ISO showed limited cytotoxicity in the tested mammalian cells and did not produce marked abnormalities in the evaluated short-term safety parameters under the present experimental conditions.

In the protoscolex survival assay, 10 μM ISO and 40 μM ABZSO both resulted in complete protoscolex death after approximately 5 days, whereas ISO at concentrations of 20 μM or higher produced a more rapid loss of protoscolex viability than the 40 μM ABZSO reference treatment. In the murine efficacy experiment, ABZ (100 mg/kg), ISO 10 mg/kg, and ISO 40 mg/kg were all associated with reduced lesion progression compared with untreated infected mice. Both ISO-treated groups also showed reduced spleen indices. These comparisons were performed using the predefined reference-treatment conditions and were not designed as exposure- or potency-matched head-to-head comparisons; therefore, they should not be interpreted as evidence of superiority of ISO over ABZ or ABZSO.

### 3.4 ISO treatment was associated with apoptosis-, proliferation-, and fibrosis-related changes in the AE lesion microenvironment

Ultrastructural observation by TEM showed that metacestodes in the untreated model group retained an intact architecture with normal nuclear morphology. In contrast, metacestodes from the ABZ (100 mg/kg), ISO 10 mg/kg, and ISO 40 mg/kg treatment groups exhibited marked mitochondrial swelling, loss or disruption of mitochondrial cristae, cytoplasmic vacuolization, dilated rough endoplasmic reticulum, and increased autophagosome-like structures in germinal layer cells (Figure 4A). In addition, collagen deposition outside the vesicles appeared more prominent in the treated groups than in the untreated group.

**FIGURE 4:**
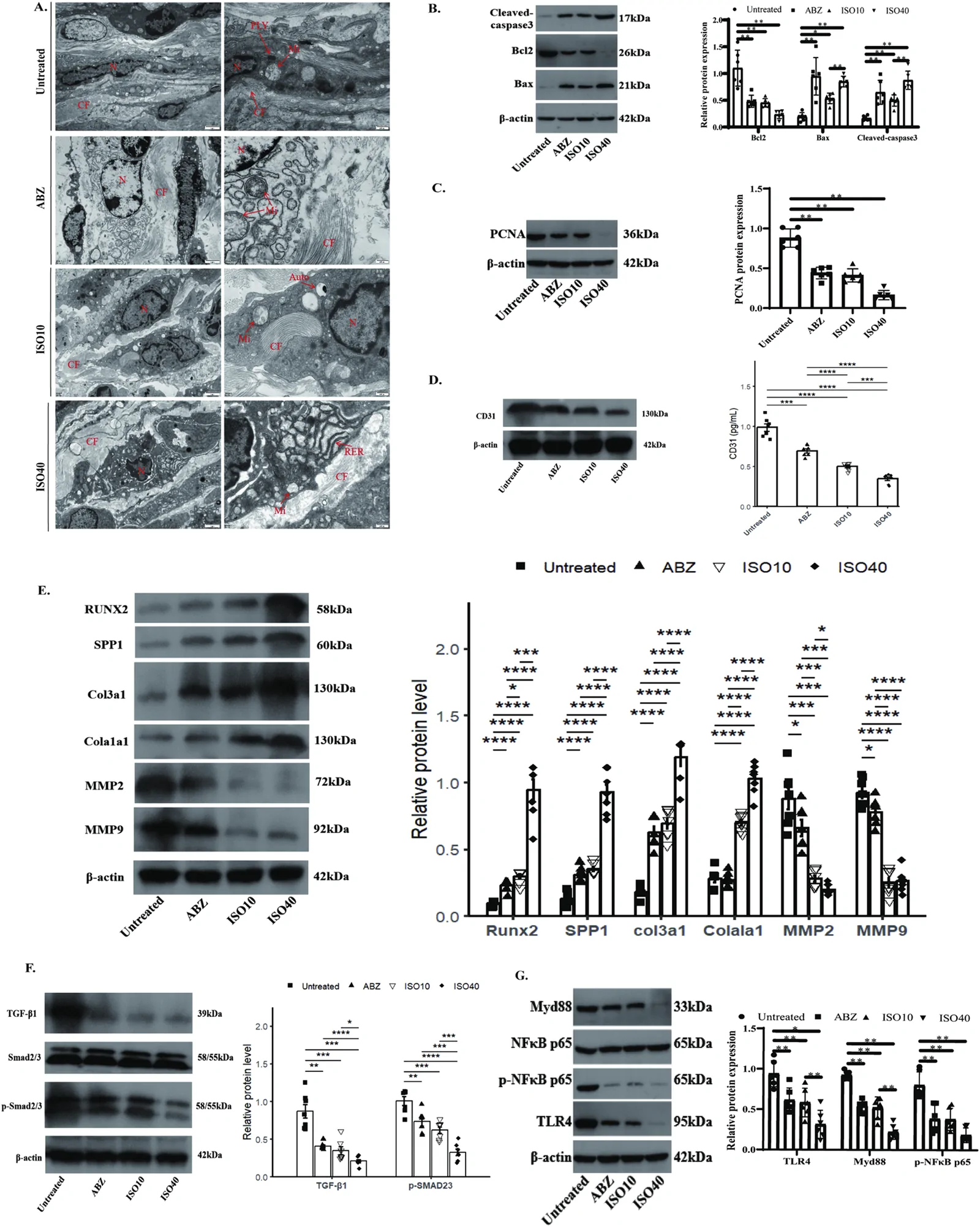
Treatment-associated protein-expression changes in the AE lesion microenvironment. **(A)** Ultrastructural changes of metacestodes observed by TEM in the untreated, ABZ (100 mg/kg), ISO 10 mg/kg, and ISO 40 mg/kg groups. Representative ultrastructural alterations included mitochondrial swelling and disruption of mitochondrial cristae, cytoplasmic vacuolization, dilated rough endoplasmic reticulum, and autophagosome-like structures. Labels indicate primary lysosomes (PLY), mitochondria (Mi), nuclei (N), collagen fibers (CF), autophagosome-like structures (Auto), and rough endoplasmic reticulum (RER). **(B)** Expression and quantitative analysis of apoptosis-related proteins (cleaved caspase-3, Bcl-2, and Bax). **(C)** Expression and quantitative analysis of PCNA. **(D)** Expression and quantitative analysis of CD31. **(E)** Expression and quantitative analysis of RUNX2, SPP1, Col1a1, Col3a1, MMP2, and MMP9. ISO treatment was associated with higher RUNX2, SPP1, Col1a1, and Col3a1 expression and lower MMP2 and MMP9 expression. **(F)** TGF-β1 expression was lower in the ISO group than in the untreated group. p-Smad2/3 was also decreased, while total Smad2/3 showed no significant change. **(G)** Expression and quantitative analysis of TLR4/NF-κB pathway-related proteins (MyD88, NF-κB p65, p-NF-κB p65, TLR4).* Indicates *p* < 0.05. ** Indicates *p* < 0.01. *** Indicates *p* < 0.001. **** Indicates *p* < 0.0001.

In host perilesional tissues adjacent to the metacestodes, Bcl-2 expression was lower and Bax and cleaved caspase-3 levels were higher in the ISO-treated groups than in the untreated group (*p* < 0.01) (Figure 4B). PCNA protein expression was also significantly decreased in all treatment groups (*p* < 0.01) (Figure 4C). In addition, CD31 expression was significantly lower in the ABZ and ISO groups than in the untreated group, with the greatest reduction observed in the 40 mg/kg ISO group (*p* < 0.01) (Figure 4D).

Further analysis of osteogenic differentiation markers showed that ISO treatment was associated with higher RUNX2, SPP1, Col1a1, and Col3a1 expression and lower MMP2 and MMP9 expression (Figure 4E). In the same tissue samples, TGF-β1 expression and p-Smad2/3 levels were lower in the ISO-treated groups than in the untreated group, whereas total Smad2/3 did not show a significant change (Figure 4F). ISO treatment also reduced TLR4 and MyD88 expression and decreased p-NF-κB p65 levels, whereas total NF-κB p65 remained relatively stable (Figure 4G).

### 3.5 ISO treatment altered T-cell subset proportions and cytokine profiles during AE infection

To characterize treatment-associated changes in the host immune response during chronic *E. multilocularis* infection, we performed flow cytometric analysis of T-lymphocyte subsets in perilesional hepatic tissues and peripheral blood, together with ELISA quantification of serum cytokine profiles, after 6 weeks of ISO or ABZ administration (n = 8 independent biological replicates per group, with each individual mouse defined as one biological replicate).

As shown in Figure 5A, ISO treatment was associated with changes in CD4^+^ and CD8^+^ T-cell proportions in both perilesional tissues and peripheral blood. Compared with untreated infected mice, mice treated with 40 mg/kg ISO showed higher proportions of CD4^+^ T cells and lower proportions of CD8^+^ T cells in both compartments, whereas the changes observed at 10 mg/kg were less pronounced. We further characterized functional CD4^+^ T-helper cell subsets in perilesional tissues (Figure 5B). Th1 cell proportions increased with increasing ISO dose, whereas Th2 and Treg proportions decreased. In contrast, Th17 cells showed a non-monotonic response, with a higher proportion in the 10 mg/kg ISO group and a lower proportion in the 40 mg/kg ISO group compared with untreated infected mice.

**FIGURE 5:**
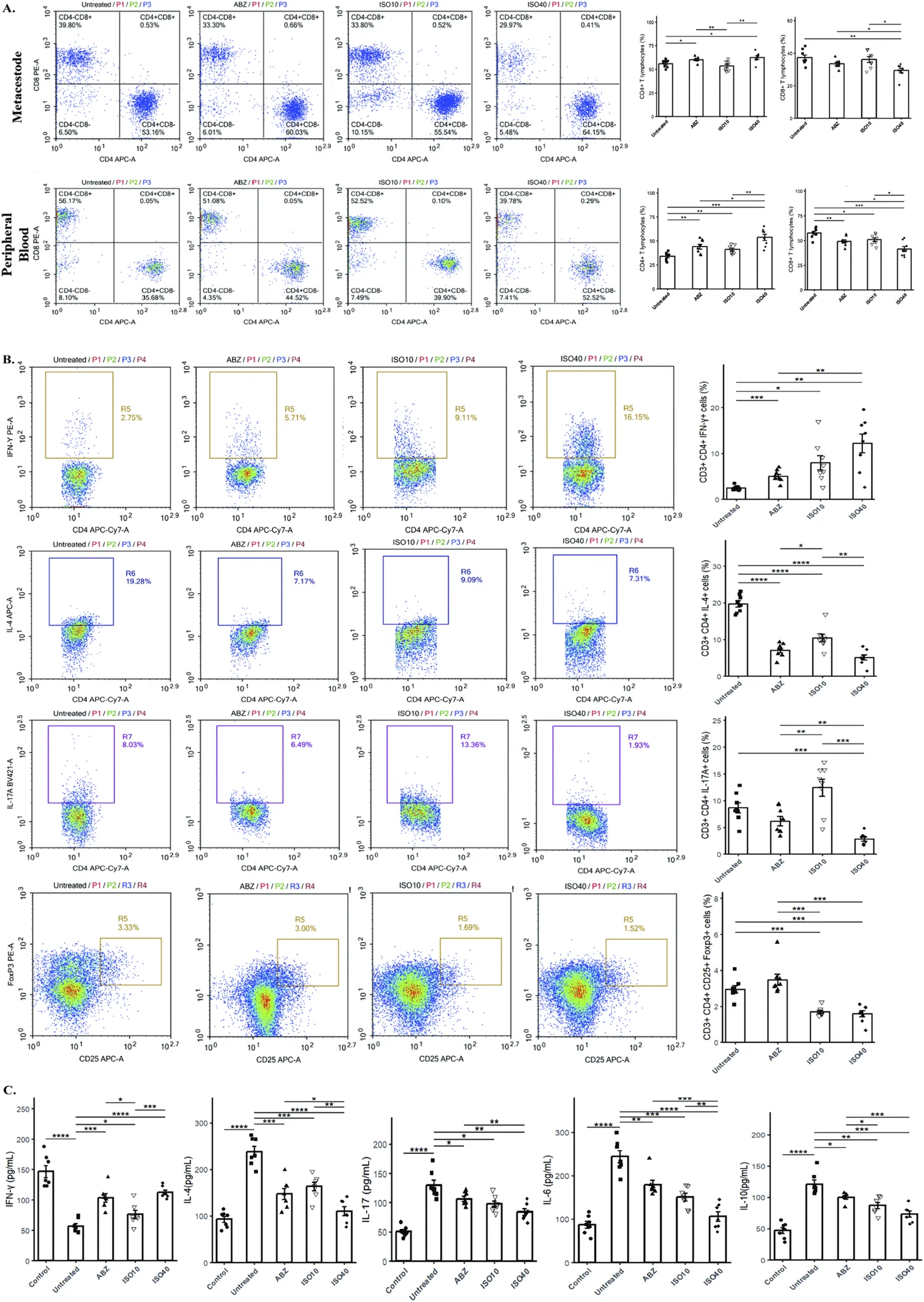
Treatment-associated changes in T-cell subsets and serum cytokines after ISO treatment. **(A)** Flow-cytometric analysis of CD4^+^ and CD8^+^ T-cell proportions in perilesional tissues and peripheral blood. **(B)** Flow-cytometric analysis of Th1, Th2, Th17, and Treg subsets in perilesional tissues. ISO treatment was associated with increased Th1 proportions and decreased Th2 and Treg proportions, whereas Th17 cells showed a non-monotonic response across ISO doses. **(C)** Changes in serum cytokines in mice after ISO intervention.* *Indicates p* < 0.05. ** *Indicates p* < 0.01. *** *Indicates p* < 0.001. **** *Indicates p* < 0.0001.

ELISA quantification of serum cytokines (Figure 5C) showed that ISO treatment dose-dependently increased IFN-γ levels and reduced IL-4, IL-10, IL-6, and IL-17 levels (*p* < 0.05 or *p* < 0.01 vs. untreated control group). Collectively, ISO treatment was associated with shifts in T-helper-cell subset proportions and altered serum cytokine profiles in infected mice.

## 4 Discussion

This study evaluated the anti-*Echinococcus multilocularis* activity of ISO using complementary *in vitro* and *in vivo* models. The principal findings were that ISO reduced protoscolex viability, increased PGI release from metacestode vesicles, reduced germinal layer cell viability, and reduced lesion progression in infected mice. Parasite-specific analyses further showed structural injury, mitochondrial ultrastructural abnormalities, mitochondrial membrane potential depolarization, and reduced β-tubulin expression following ISO treatment. In parallel, the *in vivo* experiments revealed treatment-associated changes in apoptosis-, proliferation-, fibrosis-, inflammation-, and immune-related readouts in the host lesion microenvironment.

### 4.1 Direct anti-parasitic activity of ISO and parasite-associated alterations

The present *in vitro* data provide direct evidence that ISO exerts anti-*E*. *multilocularis* activity. ISO caused concentration- and time-dependent loss of protoscolex viability, increased PGI release from metacestode vesicles, and reduced the viability of germinal layer cells. Morphological and ultrastructural analyses also demonstrated disruption of vesicle architecture and marked structural injury in protoscoleces. The combined viability and morphological data therefore indicate direct antiparasitic activity under the tested conditions.

Mitochondrial alterations were among the most prominent parasite-associated changes. TEM revealed mitochondrial swelling and disruption or loss of mitochondrial cristae, while JC-1 staining demonstrated a significant reduction in the mitochondrial membrane potential of ISO-treated protoscoleces. The concordant TEM and JC-1 results are consistent with mitochondrial perturbation after ISO exposure. Mitochondrial energy metabolism has been identified as a potentially exploitable vulnerability in *E. multilocularis*, with both aerobic electron transport and anaerobic respiratory pathways contributing to parasite energy metabolism and survival (Chaudhry et al., 2022; Enkai et al., 2020). More recently, ESI-09 was shown to disrupt mitochondrial energy metabolism by acting as a mitochondrial uncoupler in *E. multilocularis* germinal layer cells, further supporting parasite mitochondrial function as a pharmacologically vulnerable process (Zumstein et al., 2025). Similar reductions in mitochondrial membrane potential and mitochondrial ultrastructural abnormalities have also been reported following treatment with other compounds showing anti-*E. multilocularis* activity (Lu et al., 2024), but such parallels do not imply a shared molecular target or downstream cell-death pathway.

In particular, the present data are insufficient to conclude that mitochondria-dependent apoptosis mediates parasite killing. Ultrastructural morphology alone is insufficient to establish activation of a defined apoptosis pathway. Moreover, Bax, Bcl-2, and cleaved caspase-3 were measured in host perilesional tissues rather than in the parasite and therefore cannot be used as direct evidence of apoptosis signaling in *E. multilocularis*. Additional parasite-specific biochemical and molecular studies will be required to determine whether programmed cell-death pathways contribute to ISO-induced parasite injury.

ISO treatment was also associated with reduced β-tubulin expression in protoscoleces. β-Tubulin is an important component of the parasite microtubule cytoskeleton, and experimental disruption of β-tubulin expression in *E. multilocularis* protoscoleces has been associated with impaired viability and ultrastructural damage to the tegument and flame cells. Therefore, the reduced β-tubulin expression observed in the present study may be relevant to the structural alterations detected after ISO treatment. However, reduced β-tubulin expression does not establish β-tubulin as a direct molecular target of ISO and could instead represent a downstream consequence of cellular injury, metabolic disturbance, or altered parasite viability.

Similarly, the molecular docking analyses predicted potential interactions of ISO with β-tubulin and selected metabolic enzymes, but these results should be considered hypothesis-generating. Computationally predicted binding poses do not demonstrate direct target engagement under physiological conditions. Previous studies investigating drug targets in *E. multilocularis* have used experimental affinity-based approaches coupled with proteomic analyses to identify candidate drug-binding proteins, highlighting the importance of experimental target validation beyond computational prediction (Memedovski et al., 2023). Confirmation of the putative ISO–protein interactions will therefore require direct biochemical binding assays, microtubule polymerization studies, and functional validation of the predicted metabolic targets.

### 4.2 Treatment-associated changes in the AE lesion microenvironment

In addition to the direct parasite-associated effects, ISO treatment was accompanied by changes in the tissues surrounding AE lesions. The perilesional microenvironment of AE is characterized by dynamic interactions among inflammatory cells, fibroblasts, extracellular matrix components, and vascular structures, which are closely associated with lesion progression and host tissue remodeling (Liu et al., 2021a; Tian et al., 2021; Wu et al., 2025). These host responses should therefore be interpreted separately from the parasite-specific findings.

In perilesional host tissues, ISO treatment was associated with lower Bcl-2 expression and higher Bax and cleaved caspase-3 levels. The Bax, Bcl-2, and cleaved caspase-3 expression pattern was consistent with apoptosis-related changes in perilesional host tissues during treatment. Apoptotic and necrotic changes in hepatic cells have also been reported during the course of experimental *E. multilocularis* infection, indicating that host-cell death represents one component of the local pathological response to infection (Tian et al., 2021). Because Bax, Bcl-2, and cleaved caspase-3 were not measured in *E. multilocularis* in the present study, however, they should not be interpreted as evidence that ISO activates the same apoptosis-related pathways in the parasite. Whether these host-cell changes contribute to lesion remodeling or occur secondary to reduced parasite burden remains unclear.

ISO treatment was also associated with lower PCNA and CD31 expression in perilesional tissues. Reduced PCNA staining is consistent with decreased proliferative activity in the local host tissue environment, whereas reduced CD31 expression suggests changes in lesion-associated vascularization. Pathological angiogenesis has been documented around *E. multilocularis* lesions, with increased expression of CD31 and other angiogenesis-related factors in infected tissues (Bi et al., 2025; Yuan et al., 2019). Experimental inhibition of VEGF-associated angiogenesis has also been shown to restrict echinococcal lesion development, supporting a potential relationship between the perilesional vascular environment and parasite growth (Yuan et al., 2019). Because CD31 expression alone does not establish changes in neovascularization, the observed reduction should be interpreted as a treatment-associated vascular change rather than evidence of a direct anti-angiogenic effect.

Changes were observed in fibrosis- and extracellular-matrix-related readouts, but they did not show a uniformly antifibrotic pattern. ISO treatment was associated with higher RUNX2, SPP1, Col1a1, and Col3a1 expression and lower MMP2 and MMP9 expression, whereas TGF-β1 and p-Smad2/3 levels were reduced. Consistent with the increased collagen-associated protein expression, TEM also suggested more prominent collagen deposition outside the vesicles in the treated groups. Perilesional fibrosis is an important component of the host response to E. multilocularis infection, and TGF-β-associated signaling has been implicated in collagen deposition and fibrotic remodeling during AE (Tian et al., 2021; Wu et al., 2025). The discordance between the collagen- and matrix-associated readouts and the lower TGF-β1/p-Smad2/3 levels indicates that the tissue response to ISO cannot be interpreted as a simple reduction in fibrosis. These findings are therefore best regarded as treatment-associated changes in extracellular-matrix remodeling, and their relationship to lesion regression remains to be clarified.

Overall, these host-tissue changes may reflect both direct drug effects and secondary responses to reduced parasite burden; their causal contribution to treatment efficacy was not tested in the present study.

### 4.3 Treatment-associated immune and inflammatory responses

ISO treatment was accompanied by changes in inflammatory signaling, T-cell subset distribution, and cytokine profiles in infected mice. In perilesional tissues, TLR4 and MyD88 expression and p-NF-κB p65 levels were lower following ISO treatment. In parallel, ISO-treated mice showed higher proportions of Th1 cells and lower proportions of Th2 and Treg cells, whereas Th17 proportions differed between the two ISO doses, increasing at 10 mg/kg and decreasing at 40 mg/kg. Serum IFN-γ levels were higher, whereas IL-4, IL-6, IL-10, and IL-17 levels were lower after ISO treatment. The TLR4/MyD88/NF-κB signaling axis has been implicated in the inflammatory response surrounding AE lesions in both patients and experimental *E. multilocularis* infection models (Zhu et al., 2024). Experimental manipulation of MyD88/NF-κB signaling has also been shown to alter Th1/Th2 balance, inflammatory responses, liver injury, and hepatic fibrosis during AE, indicating that this pathway participates in the complex host response to infection (Wang L et al., 2025).

T-cell composition within the AE lesion microenvironment is likewise heterogeneous and disease-associated. Single-cell analysis of human AE tissues demonstrated enrichment of Th17 cells and induced regulatory T cells in perilesional liver tissue, together with expression patterns associated with immune suppression (Jiang et al., 2022). Longitudinal clinical studies have also shown that systemic cytokine and chemokine profiles vary according to progressive, stable, or cured AE disease states, further indicating that host immune readouts are closely associated with disease activity (Grüner et al., 2022).

The immune and inflammatory profiles of ISO-treated mice therefore differed from those of untreated infected mice. The observed pattern is compatible with a shift in the immune environment during treatment; however, the present study cannot determine whether ISO directly acts on host immune cells. Importantly, antiparasitic treatment itself can alter cytokine, chemokine, and T-cell-associated immune profiles in *E. multilocularis*-infected animals, as previously reported following albendazole treatment (Wu et al., 2021). Therefore, reduced parasite burden and the accompanying decrease in persistent antigenic or inflammatory stimulation may secondarily contribute to the changes in TLR4/MyD88/NF-κB signaling, cytokine production, and T-cell subset proportions observed after ISO treatment.

Accordingly, the present data should not be interpreted as demonstrating that ISO directly “restores” or “induces” protective anti-AE immunity. Rather, they indicate that ISO treatment is associated with changes in host immune and inflammatory responses during reduction of AE lesion progression. Distinguishing direct immunomodulatory effects from secondary consequences of parasite control will require experiments in which ISO is tested directly on defined host immune-cell populations, together with experimental designs that independently control for parasite burden.

The present experiments did not determine the contribution of these host responses to treatment efficacy and do not establish immune modulation as an independent mechanism of parasite killing.

### 4.4 Comparison with ABZ/ABZSO and implications for further development

ABZ remains a principal pharmacological treatment for AE, and benzimidazoles are currently the only drug class recommended for pharmacological treatment of the disease (Autier et al., 2024; Jing et al., 2024). In the present study, ABZSO was included in the *in vitro* experiments as a positive-control benchmark. In the protoscolex survival assay, 10 μM ISO and 40 μM ABZSO required approximately 5 days for complete loss of protoscolex viability, whereas higher ISO concentrations produced more rapid killing under the tested conditions. However, ISO and ABZSO were not evaluated at equivalent or equipotent concentrations. Differences in killing kinetics therefore represent an assay-specific benchmark comparison and should not be interpreted as evidence that ISO is more effective or therapeutically superior to ABZ/ABZSO. This distinction is particularly important because benzimidazoles are predominantly parasitostatic in AE, and although numerous compounds have shown activity against *E. multilocularis* experimentally, none has yet been clearly demonstrated to be more effective than benzimidazole therapy in humans (Autier et al., 2024).

The *in vivo* experiments similarly showed that both ABZ and ISO treatment were associated with reduced lesion progression. ISO also showed limited cytotoxicity in the tested mammalian cell models and no obvious overall short-term toxicity during the 6-week mouse study. In the separate toxicity experiment, hematological and biochemical parameters showed no significant differences between the ISO 40 mg/kg and control groups. These findings provide preliminary information regarding short-term tolerability but are insufficient to establish the long-term safety profile or therapeutic window of ISO. Current pharmacological reviews of ISO similarly emphasize that further preclinical characterization, including pharmacokinetic and toxicological evaluation, is required before clinical translation can be considered (Yang et al., 2024).

Direct comparison with ABZ will require exposure- or potency-matched efficacy studies together with comparative pharmacokinetic analyses. Given the limited aqueous solubility and low oral bioavailability reported for ISO (Yang et al., 2024; Zhou et al., 2018), formulation optimization and longer-term efficacy and safety evaluation are also warranted.

## 5 Conclusion

In summary, ISO showed direct anti-*E. multilocularis* activity *in vitro* and reduced AE lesion progression in an experimental mouse model. Treatment was accompanied by parasite structural and mitochondrial alterations, reduced β-tubulin expression, and changes in multiple host lesion-microenvironment readouts. These associated changes, together with the docking results, do not establish a specific causal mechanism or direct molecular target of ISO. Further preclinical evaluation of ISO should prioritize parasite-specific mechanistic studies, pharmacokinetic and formulation optimization, and longer-term efficacy and safety assessment.

## Data Availability

The original contributions presented in the study are included in the article/Supplementary Material, further inquiries can be directed to the corresponding author.

## Supplementary material

SUPPLEMENTARY FIGURE S1 Two-step screening of small-molecule compounds with anti-protoscolex activity. Forty prioritized small-molecule compounds were first tested against protoscoleces at a final concentration of 50 μM for 3 days, and protoscolex viability was assessed by eosin staining. Oxyclozanide, Quinacrine Dihydrochloride Dihydrate, Milbemycin Oxime, Isoalantolactone, Robenidine Hydrochloride, Resorantel, Rafoxanide, and Selamectin induced complete protoscolex death under these conditions. These 8 candidate compounds were then further evaluated at a final concentration of 20 μM for 3 days, and only Isoalantolactone resulted in complete protoscolex death. Therefore, Isoalantolactone was selected for subsequent studies.

SUPPLEMENTARY FIGURE S2 Chemical structure of isoalantolactone (ISO). Two-dimensional and three-dimensional structural representations of isoalantolactone are shown. ISO is a sesquiterpene lactone of the eudesmanolide type and has been reported as a natural product isolated from Inula helenium and other plant species. This figure is provided to illustrate the chemical structure of the compound investigated in this study. Source: PubChem, isoalantolactone, available at: https://pubchem.ncbi.nlm.nih.gov/.
